# Metabolic responses of willow (*Salix purpurea* L.) leaves to mycorrhization as revealed by mass spectrometry and ^1^H NMR spectroscopy metabolite profiling

**DOI:** 10.3389/fpls.2015.00344

**Published:** 2015-05-18

**Authors:** Konstantinos A. Aliferis, Rony Chamoun, Suha Jabaji

**Affiliations:** Department of Plant Science, McGill UniversitySainte-Anne-de-Bellevue, QC, Canada

**Keywords:** arbuscular mycorrhizal fungi, metabolomics, plant-fungal interactions, plant selection, plant stress responses

## Abstract

The root system of most terrestrial plants form symbiotic interfaces with arbuscular mycorrhizal fungi (AMF), which are important for nutrient cycling and ecosystem sustainability. The elucidation of the undergoing changes in plants' metabolism during symbiosis is essential for understanding nutrient acquisition and for alleviation of soil stresses caused by environmental cues. Within this context, we have undertaken the task of recording the fluctuation of willow (*Salix purpurea* L.) leaf metabolome in response to AMF inoculation. The development of an advanced metabolomics/bioinformatics protocol employing mass spectrometry (MS) and ^1^H NMR analyzers combined with the in-house-built metabolite library for willow (http://willowmetabolib.research.mcgill.ca/index.html) are key components of the research. Analyses revealed that AMF inoculation of willow causes up-regulation of various biosynthetic pathways, among others, those of flavonoid, isoflavonoid, phenylpropanoid, and the chlorophyll and porphyrin pathways, which have well-established roles in plant physiology and are related to resistance against environmental stresses. The recorded fluctuation in the willow leaf metabolism is very likely to provide AMF-inoculated willows with a significant advantage compared to non-inoculated ones when they are exposed to stresses such as, high levels of soil pollutants. The discovered biomarkers of willow response to AMF inoculation and corresponding pathways could be exploited in biomarker-assisted selection of willow cultivars with superior phytoremediation capacity or genetic engineering programs.

## Introduction

Willow (*Salix* spp.) is a highly diverse genera containing fast growing species used for biomass production (Labrecque and Teodorescu, 2003; Djomo et al., 2015), bioenergy and biofuels (Karp et al., 2011), phytoremediation (Guidi et al., 2012), and erosion control (Bariteau et al., 2013). This diversity is mainly due to willows' unique characteristics such as, superior growth rate and extensive fibrous root system, and adaptability to extreme environmental and soil conditions (Jensen et al., 2009; Vangronsveld et al., 2009). The latter is improved by its symbiotic relationship with obligate biotrophs, the arbuscular mycorrhizal fungi (AMF), present in its rhizosphere (Bamforth and Singleton, 2005; Bonfante and Genre, 2008, 2015; Leigh et al., 2009). AMF are important components of ecosystems forming symbiotic relationships with the roots of the vast majority of plants (Smith and Read, 2008), which contribute to their improved nutrition and stress tolerance, and enhance soil structure (van der Heijden et al., 2006; Vogelsang et al., 2006).

The impact of AMF on willow has been investigated at different levels, however, there is no information on their effect on willows' global metabolism regulation. Increased root length and shoot growth in *Salix repens* has been reported following colonization by the AMF *Glomus mosseae* (van der Heijden, 2001). Also, colonization of *Salix miyabeana* and *Salix viminalis* by *Glomus intraradices* has resulted in increased phosphorus content in stems providing advantages for phytoremediation of heavy metals due to increased biomass (Fillion et al., 2011). The latter has been investigated on the ability of *G. intraradices*-*S. viminalis* interaction to rehabilitate a disturbed and slightly contaminated brownfield (Bissonnette et al., 2010).

AMF colonization is restricted to the root system, however, its effects are detectable, even macroscopically, in the above-ground plant parts (Smith and Read, 2008). New evidence is emerging on the capability of AMF on regulating plant genes involved in metabolic processes such as, defense and hormonal metabolism in shoots and leaves (Fiorilli et al., 2009; Lopez-Raez et al., 2010; Zouari et al., 2014). In addition to the impact on plant growth and resistance, mycorrhization improves the nutritional quality of fruits and leaves of agricultural crops via increased levels of plant secondary metabolites (Toussaint et al., 2007; Baslam et al., 2011), which are important for enhanced plant tolerance to stresses (Jeffries et al., 2003).

Metabolomics for the study of willow is still in its infancy with a handful of studies focusing on the phytochemical properties of willow bark and leaves (Du et al., 2007; Förster et al., 2010; Agnolet et al., 2012), the inhibitory compounds in lignocellulosic willow wood chips hydrolysates (Zha et al., 2014), and the chemical composition of the cuticular wax in relation to biomass productivity (Teece et al., 2008). These studies report on the accumulation of primary and secondary metabolites in the aerial parts of AMF willows. To our knowledge, there are no studies relevant to the effect of the AMF symbiosis on the leaf metabolome of willows, which highlights the need for further research on the mechanism by which it affects willows' metabolism and metabolite allocation to above-ground plant parts.

Within this context, as part of a multidisciplinary research project (GenoRem, http://genorem.ca) aiming at the optimization of willow-AMF symbiosis for phytoremediation purposes, we have undertaken the task of dissecting the effect of AMF on young willows' (*Salix purpurea* L. cv Fish Creek) leaf metabolism. Results will reveal the significance of AMF on willow's metabolism, and its indirect correlation with their phytoremediation capacity, when the willow-AMF system is a component of integrated phytoremediation strategies. For example, up-regulation of certain biosynthetic pathways as a result of AMF colonization could impact the adaptability of the plant and its performance under unfavorable conditions. To achieve this task, an advanced metabolomics/bioinformatics protocol was established employing proton nuclear magnetic resonance spectroscopy (^1^H NMR), gas chromatography-mass spectrometry (GC/MS), and liquid chromatography-MS (LC/MS) using an LTQ Orbitrap analyzer for the monitoring of the global metabolism regulation of willow in response to AMF colonization. An essential element of the study was the development of a metabolite species-specific library for willow, which accelerated the steps of metabolite identification and biological interpretation of results.

## Materials and methods

### Chemicals and reagents

Chemicals and reagents used for GC/MS sample derivatization [i.e., methoxylamine hydrochloride 99.8%, *N*-methyl-*N*-(trimethyl-silyl)trifluoroacetamide (MSTFA) 98%, and pyridine] and for ^1^H NMR analysis [i.e., deuterium oxide 99.9% (D_2_O) containing 0.05% trimethylsilyl-2,2,3,3-d4-propionic acid sodium salt (TSP)] were purchased from Sigma-Aldrich Canada Ltd. (Oakville, ON, Canada). Ethyl acetate, methanol, formic acid, ammonium acetate (Optima grade®), and water (HPLC grade) were purchased from Fisher Scientific Company (Ottawa, ON, Canada). The ProteoMass ESI Calibration Kit MSCAL5 and MSCAL6 (Sigma-Aldrich), and the DRO/GRO Range Calibration Standard (Restek Corporation, Bellefonte, PA, USA, catalog #31832), which is a mixture of 12 alkanes, were used for the calibration and monitoring instruments' performance. The peptide leucine-encephalin (Sigma-Aldrich) was used as internal standard in LC/MS analysis.

### Biological material and inoculation of willows with *Rhizophagus irregularis*

Experiments were conducted in the greenhouse of the Institut de Recherche en Biologie Végétale (IRBV) (Montreal, Canada) following a completely randomized design and under controlled conditions; temperature of 20°C at day/18°C at night, relative humidity of 50%, and light intensity of 300 μE/m^2^ s for 16 h per day. The AMF *Rhizophagus irregularis* isolate DAOM-240415 was obtained from the Canadian National Mycological Herbarium (Ontario, Canada) and maintained *in vitro* on modified minimal media (MM) (Bécard and Fortin, 1988) solidified with 0.4% (w/v) gellan gum (Sigma) with carrot roots transformed with Ri T-DNA of *Agrobacterium rhizogenes*. For simplicity from here and onwards the term AMF will be used instead of *R. irregularis*. The inoculum suspension was prepared from mycelia, spores and roots harvested from MM, dissolved in extraction buffer (0.82 mM sodium citrate and 0.18 mM citric acid) and mixed in a blender for 30 s (Hijri and Sanders, 2004). Willow cuttings (*S. purpurea* cv. Fish Creek) of 20 cm length were planted in pots containing an autoclaved mixed substrate composed of peat-moss, sand, and soil (1:1:1, v/v/v) with available P inferior to 10 mg m^−3^ for 6 weeks. Subsequently, seedlings were individually inoculated with 5 mL of inoculum suspension (approximately 500 propagules) whereas 5 mL of autoclaved water were added to non-mycorrhizal control plants that were placed at a distance from the inoculated ones to reduce the possibility of cross-contamination. No fertilizers or amendments were used. Plants were watered to soil capacity with deionized water every 2 days and were harvested 2 weeks following AMF inoculation when stems had reached approximately 1 m in length. There were five plants per treatment, each in a separate pot. Mycorrhizal colonization for willow roots was confirmed by microscopic observation of stained root sections (Vierheilig et al., 1998). The rate of the endo-mycorrhizal colonization was around 6% of root length, a level of colonization reported for willows (Bissonnette et al., 2010).

### Sampling and metabolite extraction

The top five fully expanded leaves were harvested per plant. For MS analyses, two plugs were taken from each leaf, and the plugs were pooled (approximately 72 mg of fresh weight). Sample extraction and processing was performed as previously described (Aliferis et al., 2014) using 1 mL of a mixture of methanol:ethyl acetate (50:50, v/v). Following filtering, they were divided into two portions of 0.5 mL in glass autosampler vials for GC/MS and LC/MS analyses. The latter was further divided into two portions (0.25 mL each) for analysis in positive (ESI^+^) and negative (ESI^−^) electrospray modes. For GC/MS, samples were spiked by adding 20 μL of a ribitol solution (0.2 mg mL^−1^) in methanol-water (50:50, v/v). Finally, extracts were dried using a Labconco CentriVap refrigerated vacuum concentrator (Labconco, Kansas City, MO, USA) equipped with a cold trap.

For ^1^H NMR analyses, pulverized leaf material (100 mg) was lyophilized for 24 h and dissolved in D_2_O (1 mL) for the extraction of polar compounds in glass autosampler vials (2 mL). Extracts were sonicated for 25 min and kept under continuous agitation (150 rpm) for 1 h at 24°C. For the removal of debris, samples were centrifuged (12,000 × *g*) for 1 h and the supernatants were subjected to a second centrifugation (12,000 × *g*) for 30 min. Supernatants were then collected and kept at −80°C until the acquisition of ^1^H NMR spectra.

### Chemical analyses and data pre-processing

#### Gas chromatography-mass spectrometry (GC/MS)

Derivatization of samples for GC/MS analyses was performed as previously described (Aliferis and Jabaji, 2012; Aliferis et al., 2014). Briefly, methoxymation was performed using methoxylamine hydrochloride (80 μL, 20 mg mL^−1^ in pyridine) to the dried extracts, incubated for 120 min at 30°C, followed by silylation using MSTFA (80 μL, 37°C for 90 min). Derivatized samples were transferred to microinserts (150 μL, Fisher Scientific Company), which were placed in glass autosampler vials (2 mL). An Agilent 7890A GC platform (Agilent Technologies Inc. Santa Clara, CA, USA) equipped with a 7693A series autosampler and coupled with a 5975C series mass selective detector (MSD) was employed. Chromatogram acquisition and data pre-processing were carried out with the Agilent MSD Chemstation (v. E.02.00.493). The electron ionization was set at 70 eV and full scan mass spectra were acquired at the mass range of 50–800 Da at 1 scan s^−1^ rate with a 10 min solvent delay. The temperatures were; ion source at 150°C, transfer line at 230°C, and injector at 230°C. Samples (1 μL) were injected using a split ratio of 10:1 into a HP-5MS ultra inert (UI) capillary column (30 m × 250 μm I.D., 0.25 μm film thickness; Agilent Technologies Inc.). Helium was used as the carrier gas (flow rate 1 mL min^−1^). The temperature of the oven was 70°C stable for 5 min, followed by a 5°C min^−1^ increase to 310°C and finally stable for 1 min.

#### Liquid chromatography-mass spectrometry (LC/MS)

The dried samples were re-dissolved in 160 μL of a mixture of methanol:formic acid (0.1% v/v) (50–50, v/v) or 160 μL of methanol:ammonium acetate (2.5 mM) for analysis in ESI^+^ and ESI^−^, respectively. Extracts were then transferred to microinserts and placed in glass autosampler vials. An LTQ-Orbitrap MS Classic (Thermo Scientific, San Jose, CA, USA), equipped with a reverse phase Luna®C18(2) column (cat. no. 00F-4251-B0, 150 × 2.0 mm, 3 μm, 100 Å pore size) (Phenomenex, Torrance, CA, USA) and a Security Guard Cartridge (cat. no. KJO-4282, Phenomenex), was used. All experimental events were controlled by the software Xcalibur v.2 (Thermo Scientific). Specifications of the analyzer have been described previously (Aliferis et al., 2014). Samples (10 μL) were injected manually at a flow rate of 1 μL min^−1^ using a syringe (Hamilton, Reno, NV, USA). The gradients used for ESI^+^ and ESI^−^ are displayed in the Supplementary Tables 1, 2, and settings of the LTQ Orbitrap MS in the Supplementary Tables 3, 4, respectively.

Analyses were performed at a mass resolution of 60,000 at *m/z* 400 and spectra were acquired over the range of 80–1200 Da. For selected samples, MS/MS analyses were performed with the normalized collision energy maintained at 35 eV, the activation q set to 0.25 and the activation time to 30 ms. Target ions already selected for MS/MS were dynamically excluded for 15 s. Acquired chromatograms (^*^.raw) were processed using the software SIEVE v.2.0 (Thermo Scientific) after setting optimization for ESI^+^ and ESI^−^ (Supplementary Tables 5, 6).

#### Nuclear magnetic resonance (^1^H NMR) spectroscopy

^1^H NMR spectra were recorded using a Varian Inova 500 MHz ^1^H NMR spectrometer (Varian, Palo Alto, CA, USA) equipped with a ^1^H (^13^C,^15^N) triple resonance cold probe as previously described (Aliferis and Jabaji, 2010). A total of 128 transients of 64 K data points were acquired per sample with a 90° pulse angle, 2 s acquisition time, and 2 s recycle delay with presaturation of H_2_O during the recycle delay. Spectra were Fourier transformed, and the phase and baseline was automatically corrected. Offsets of chemical shifts were corrected based on the reference signal of TSP (0.00 ppm). Processing was performed using the Spectrus Processor and C+H NMR Predictor and Database v.12.01 of ACD Labs (Advanced Chemistry Development, Inc., ACD/Labs, Toronto, Canada).

### Quality control of metabolomics analyses

Standard operating procedures (SOP) and quality control (QC) measures were followed throughout the experimental steps to ensure the quality and validity of analyses. For each treatment, a QC sample was obtained by pooling aliquots of the five biological replications. Additionally, blank samples were analyzed for the detection of possible sources of contamination during the different experimental steps, such as impurities of glassware, reagents, column bleeding, or source contamination. For this purpose, blank samples were processed alongside the experimental samples and were subjected to identical handling. Detected features not related to the biological material being analyzed were excluded from analyses.

To maintain instruments' performance, calibration of the analyzers was performed following the recommended manufacturers' procedures and using calibration solutions. For GC/MS analysis, tuning of the MS detector was performed automatically using the AutoTune function and the DRO/GRO Range Calibration Standard was injected every 10 samples to monitor the performance of the instrument. Additionally, samples were spiked with ribitol in order to monitor possible shifts in retention time and the reproducibility of analyses. For LC/MS analysis, the ProteoMass ESI Calibration Kit (Sigma-Aldrich) was used to cover the range between 138 and 1822 Da for ESI^+^ analyses (catalog # MSCAL5), and between 265 and 1880 Da for ESI^−^ analyses (catalog # MSCAL6). Samples were spiked with the peptide leucine-encephalin in order to monitor shifts in retention time, the performance of the analyzer, and mass errors.

In addition to QC samples, technical replications of randomly selected samples were performed in order to access the reproducibility of analytical conditions. Samples were analyzed in completely randomized order to avoid possible variability caused by inconsistent performance of the analyzers.

### Construction of a species-specific metabolite library for willow

For high-throughput untargeted metabolomics, the use of species-specific metabolite libraries is necessary for the robust deconvolution of the vast amount of the obtained information and the decrease of false discovery rate. Although for many plant species there are comprehensive metabolite libraries (e.g., collection of PlantCyc), for willow such library does not exist. Therefore, we have undertaken the task of developing a species-specific library for willow (Willow MetaboLib v.1.0., http://willowmetabolib.research.mcgill.ca/index.html). For its construction, results from analyses and information from the metabolite libraries of PoplarCyc (http://pmn.plantcyc.org/POPLAR/class-instances?object=Compounds), Kyoto Encyclopedia of Genes and Genomes (KEGG) (http://www.genome.jp/kegg/), PubChem (http://pubchem.ncbi.nlm.nih.gov/), KNapSack (http://kanaya.naist.jp/KNApSAcK/), the European Bioinformatics Institute (EMBL-EBI) (http://www.ebi.ac.uk/), and the literature were retrieved and integrated.

### Metabolite identification

For GC/MS and NMR analysis, the identification of the vast majority of metabolic features was performed at levels 1 (absolute identification) and 3 (tentative identification), whereas for LC/MS analysis, identification was performed at levels 2 (tentative identification) and 3 (tentative identification of compound class) for the majority of metabolic features (Dunn et al., 2013), as described below (Supplementary Data set 1).

For GC/MS analysis, mass spectra searches were performed against the library of the National Institute of Standards and Technology, NIST 08 (Gaithersburg, MD, USA). Following the guidelines of the metabolomics standards initiative (MSI) (Sumner et al., 2007), selected metabolites were absolutely identified based on fragmentation patterns and retention times (RT) of authentic chemical standards analyzed on the same GC/MS system with the same analytical method. Tentative identification was performed for metabolites with a very good fit (>90%).

For ^1^H NMR, identification of metabolites was performed by comparing the recorded chemical shifts and *J*-coupling values to those of analytical standards. Additionally, identification was performed by assigning signals to corresponding metabolites using the ACD/C+H NMR Predictor and Database v.12.01 (ACD/Labs). Using the software, ^1^H NMR spectra of metabolites can be simulated and their similarities regarding chemical shifts and *J*-coupling values can be used for the identification of unknowns.

Due to its superior analytical capabilities, LTQ Orbitrap MS represents an excellent analyzer for metabolomics. However, the vast amount of obtained information is challenging even when achieving very low mass errors (e.g., <1 ppm). Here, a biologically-driven approach was employed having as main pillar the use of the Willow MetaboLib v.1.0. Putative identification of metabolites was based on targeted searches against the library taken into consideration mass accuracy, and when available, isotope and MS/MS fragmentation patterns.

Finally, to ensure the validity of analyses and metabolite identification, results of metabolite identification and their fluctuation between the two treatments from the different analyzers were cross-validated.

### Biomarker discovery

Following peak deconvolution and integration, GC/MS data were exported to Microsoft® Excel, aligned, and normalized against ribitol for the construction of the data matrix. The data matrix was then exported to SIMCA-P^+^ v.12.0.1 (Umetrics, MKS Instruments Inc. Andover, MA, USA) for the detection of trends and corresponding metabolite-biomarkers performing multivariate analyses as previously described (Aliferis and Jabaji, 2012; Aliferis et al., 2014). Principal component analysis (PCA) was initially performed for the overview of the data set and the detection of possible outliers, which could have a leverage on the analysis. The discovery of biomarkers was based on scaled and centered partial least square-discriminant analysis (PLS-DA) regression coefficients (*P* < 0.05), since by PCA the largest sources of variation may not be represented by the computed principal components (PCs). Standard errors were calculated using Jack-knifing (95% confidence interval) (Efron and Gong, 1983). The performance of the obtained model was assessed by the cumulative fraction of the total variation of the *X*'s that could be predicted by the extracted components [*Q*^2^*_(cum)_*] and the fraction of the sum of squares of all *X*'s (*R*^2^*X*) and *Y*'s (*R*^2^*Y*) explained by the current component. Additionally, GC/MS data were subjected to One-Way ANOVA performing the Student's *t*-test (*P* < 0.05) using the software JMP 8.0 (SAS Institute Inc., NC, USA).

^1^H NMR spectra were automatically aligned and bucket integrated using a 0.01 ppm bucket width and the Intelligent Bucketing function of the software Spectrus Processor with a width looseness of 50%. For the discovery of biomarkers performing ^1^H NMR analyses, the processed data were exported to Microsoft® Excel and the obtained data matrix was finally exported to SIMCA-P+ v.12.0.1 for multivariate analysis. PCA was initially performed for the overview of the data set and the detection of possible outliers. The discovery of biomarkers was performed similarly to that of GC/MS data and as previously described (Aliferis and Jabaji, 2010).

For LTQ-Orbitrap MS acquired data, the frames (rectangular regions m/z vs. RT) obtained using the software SIEVE v.2.0 were filtered for isotopes, retaining only the ions corresponding to ^12^C (Aliferis et al., 2014). In a second step, based on their coefficient of variation distribution (CV) in the reconstructed ion chromatograms (RIC), frames were filtered, retaining those with CV < 0.8. Ion intensities were normalized against the total ion current (TIC). The obtained matrix was exported to Microsoft® Excel and finally to SIMCA-P+ v.12.0.1 for multivariate analysis for the detection of trends and an overview of the analysis. In contrast to GC/MS and ^1^H NMR data, for the high-throughput analysis and biological interpretation of data, the discovery of biomarkers was performed using the integrated into SIEVE v.2.0 Student's *t*-test (*P* < 0.05).

### Metabolic networking

The global willow metabolome and biomarkers implicated in its interaction with AMF were visualized using the software Cytoscape v.2.8.2 (Smoot et al., 2011) following previously described approach (Aliferis and Jabaji, 2012; Aliferis et al., 2014). For the *ab-initio* construction of networks, the biosynthetic pathways of KEGG were used.

## Results and discussion

### The willow metabolite library “Willow Metabolib v.1.0”

**Figure 1:**
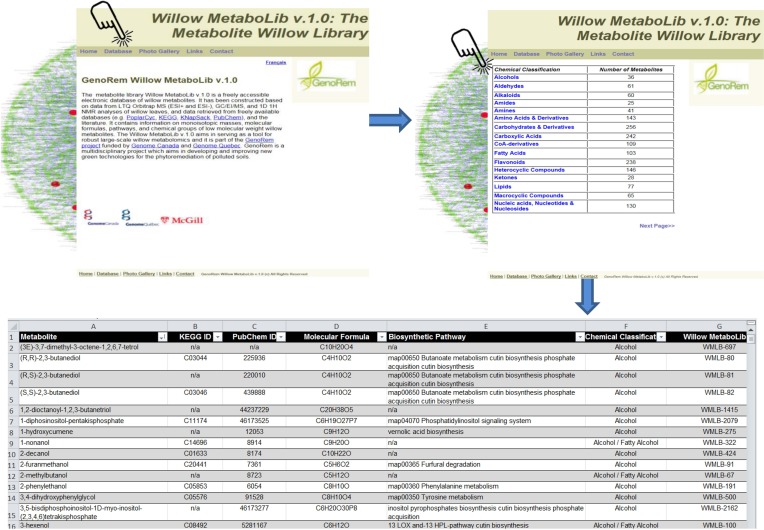
**The Willow Metabolite Library “Willow MetaboLib v.1.0” (http://willowmetabolib.research.mcgill.ca/index.html)**. In-house built metabolite library for willow containing more than 2000 entries with information on molecular formulae, standardized chemical classification, KEGG and PubChem identifiers, and biosynthetic pathways following the KEGG coding system. Sets of metabolites can be downloaded in MS Excel® format by first selecting the tab “Database” and then the desired chemical group. The website contains also other useful information on the project and links.

The application of metabolomics for the study of plant symbiosis with AMF could provide insights that can be exploited in various fields of science (Fiorilli et al., 2009). Here, for the robust deconvolution of willow's metabolome, an in-house built library for willow, the Willow MetaboLib v1.0 (http://willowmetabolib.research.mcgill.ca/index.html), was constructed. It contains more than 2000 entries with information on molecular formulae, standardized chemical classification, KEGG and PubChem identifiers, and biosynthetic pathways following the KEGG coding system (Figure 1). Metabolites are categorized based on their chemical group, and data sets can be downloaded in MS Excel format (.xls) by selecting from the home page the tab “Database,” and then the desired chemical group. The website additionally contains information on the related research, photos, and useful links. The library was used for the high-throughput identification of metabolites in the analyzed samples performing LC/MS analyses, and for cross-validation of metabolite identities performing GC/MS and ^1^H NMR analyses. Such approach is necessary toward the standardization of large-scale metabolomics data deconvolution, reporting, and biological interpretation.

### Overview of the analysis

**Figure 2:**
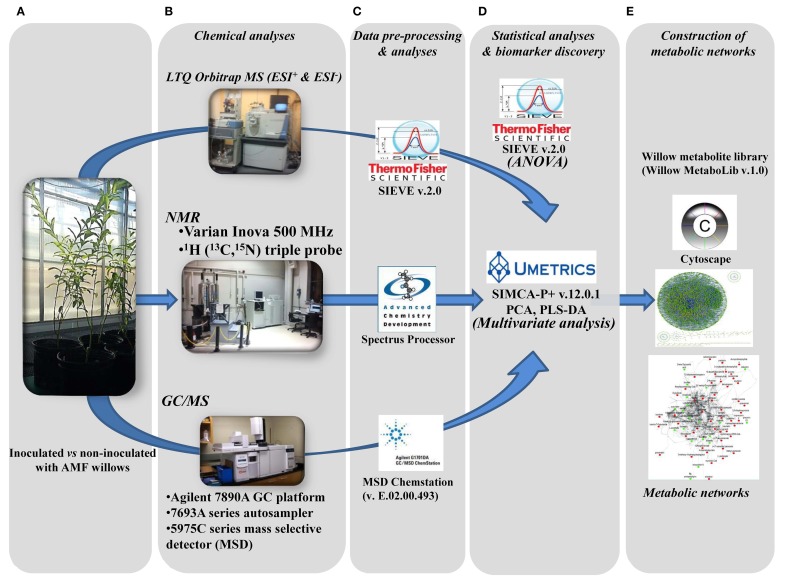
**Developed pipeline for the discovery of willow's metabolic biomarkers following inoculation with the AMF *Rhizophagus irregularis***. After sampling **(A)**, four subsequent steps were applied: **(B)** chemical analyses by integrating three analytical platforms, **(C)** data pre-processing and analyses, **(D)** statistical analyses and biomarker discovery, and **(E)** construction of metabolic networks, for the global visualization of changes in the global metabolic network of willow in response to mycorrhization.

For untargeted large-scale plant metabolomics, validated, high-throughput metabolite identification and biomarker discovery are challenging tasks. Here, the complementary capabilities of three of the most powerful and commonly employed analyzers in metabolomics (Dunn and Ellis, 2005; Kim et al., 2010) were exploited in order to accelerate and strengthen metabolite identification and expand the metabolome coverage, in combination with the use of the Willow MetaboLib v1.0 (Figures 1, 2). The robustness of the developed bioanalytical protocols is confirmed by the quality of the obtained GC/MS (Supplementary Figure 1) and LTQ Orbitrap MS (Supplementary Figure 2) chromatograms and ^1^H NMR spectra (Supplementary Figure 3).

GC/MS and ^1^H NMR were employed in order to monitor mainly the fluctuation in the levels of primary metabolites caused by AMF inoculation. A very good correlation is observed between the results obtained by the two platforms with similar fluctuation patterns in the levels of the commonly identified metabolites (Supplementary Figure 3 and Supplementary Data set 1). For example, NMR analysis revealed higher levels of sugars (Supplementary Figure 3, region 4.6–5.6 ppm) and lower levels of glutamine and pyroglutamate in AMF-inoculated willows compared to non-inoculated ones (Supplementary Figure 3, region 2.0–2.4 ppm), which is in agreement with results of GC/MS analysis (Supplementary Data set 1). In addition, NMR analysis revealed higher levels of aromatic metabolites in AMF-inoculated willows (Supplementary Figure 3, region 6.3–8.4 ppm). This is in agreement with results of LTQ Orbitrap analysis, which show high levels of flavonoids and phenolics. Additionally, performing LTQ Orbitrap analysis, fluctuation in secondary metabolites not detected/identified by GC/MS or NMR analysis such as, flavonoids, macrocyclic compounds, and phenolics, was recorded (Supplementary Data set 1). The above, justifies the notion of employing more than one analyzer for a comprehensive coverage of plant metabolism, the study of its regulation, and for strengthening our metabolite identification capacity through cross-validation (Aliferis and Jabaji, 2010; Aliferis et al., 2014).

**Figure 3:**
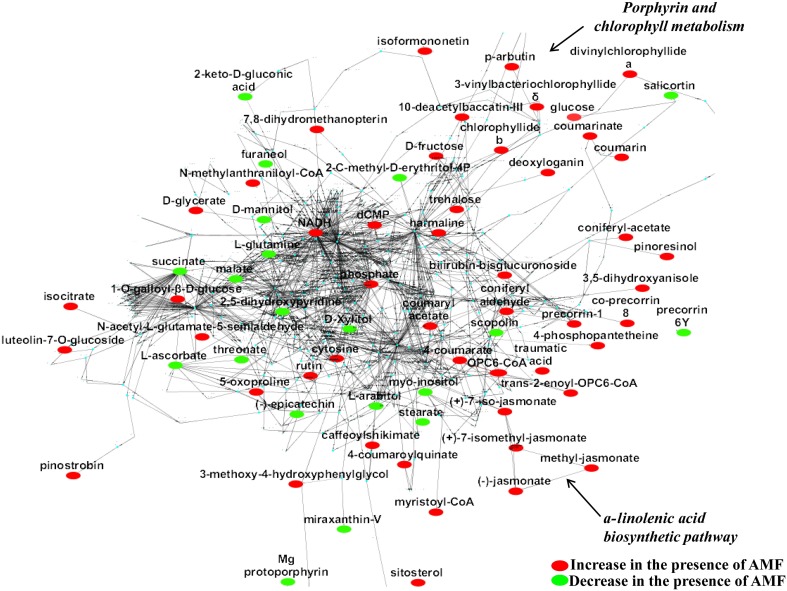
**The global willow leaf metabolic network and its perturbation 2 weeks following inoculation with the AMF *Rhizophagus irregularis* visualized using the software Cytoscape**. Biomarkers of the symbiosis and possible interconnecting networks between them are displayed. Sections of the *a*-linolenate biosynthetic pathway and porphyrin and chlorophyll metabolism are indicated by arrows. Metabolite fluctuations are displayed using a color code based on *P*-values (*P* < 0.05) performing the Student's *t*-test.

**Figure 4:**
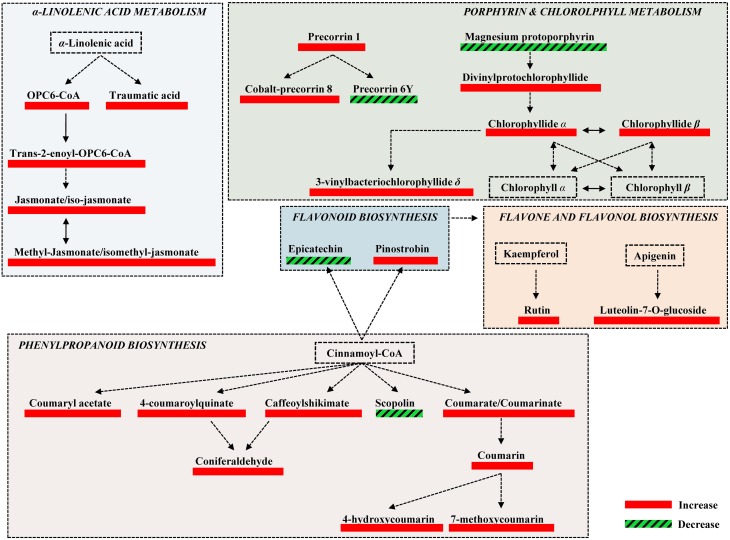
**Fluctuations of major biosynthetic pathways of willow leaves 2 weeks post-inoculation with the AMF *Rhizophagus irregularis* infection based on changes in the relative concentration of identified metabolites**. Metabolite fluctuations are displayed using a color code based on *P*-values (*P* < 0.05) performing the Student's *t*-test. Five biological replications and a quality control sample were analyzed per treatment. Dashed lines symbolize multi-step or not fully elucidated reactions and solid lines one-step reactions.

By integration of data from MS analyzers and confirmation by ^1^H NMR data, 177 significantly up- or down-regulated features were assigned to metabolites or to unique molecular formulae designated as biomarkers in response to AMF inoculation (Supplementary Data set 1) (*P* < 0.05). These biomarkers belong to various chemical groups and biosynthetic pathways of willow (Table 1). In contrast, approximately 250 identified metabolites or unique molecular formulae did not statistically differ between treatments (*P* < 0.05) (Figures 3, 4).

**Table 1:**
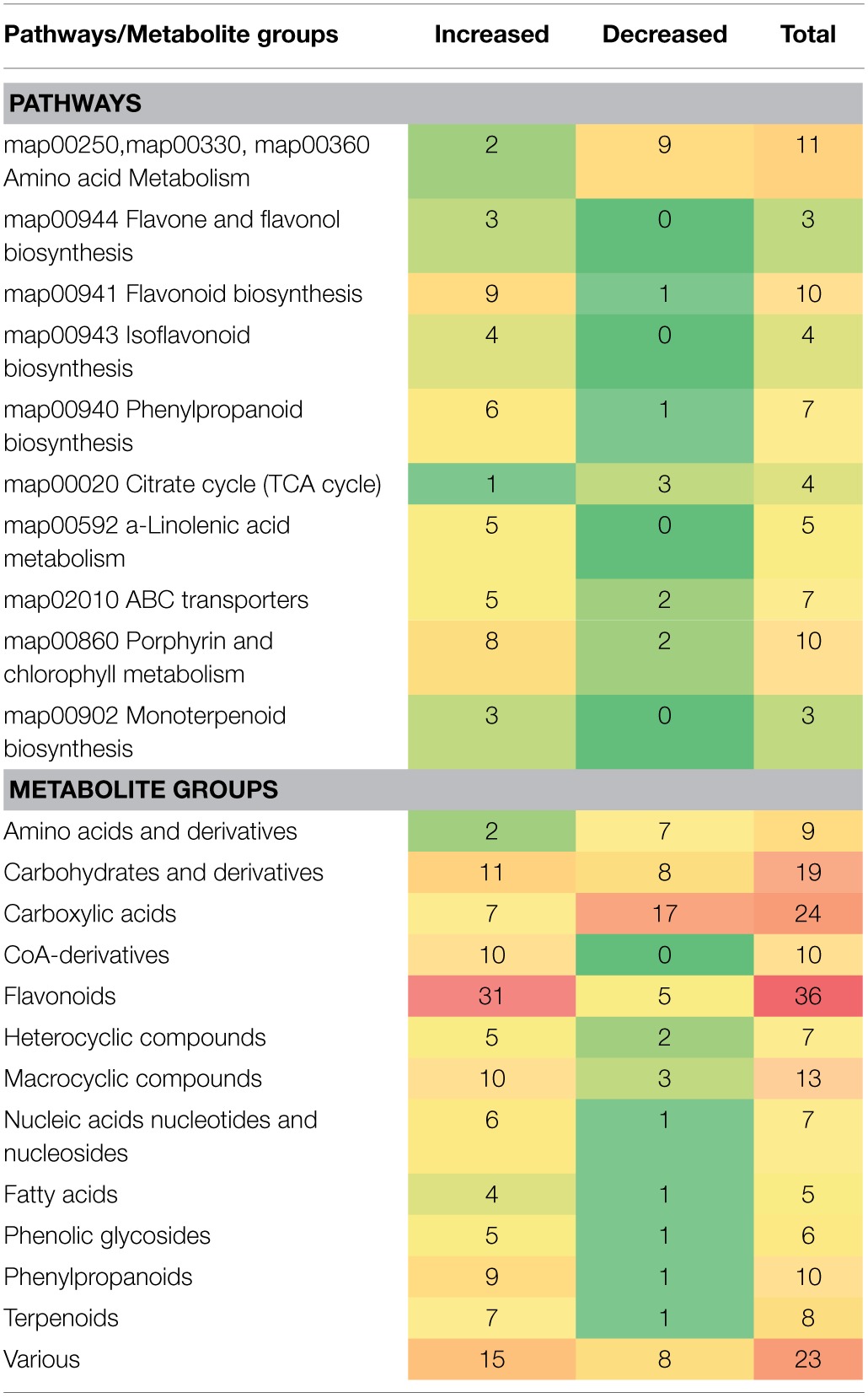
**Classification of metabolite-biomarkers of willow's leaves in response to the AMF *Rhizophagus irregularis* according to their participation in metabolic pathways/functions and chemical groups**.

*Biomarkers are classified as increased and decreased in colonized seedlings compared to controls. The code system of KEGG for pathways is used whereas for the chemical classification of metabolites information was retrieved from the database PubChem. A color gradient was used ranging from dark green (0) to red (36)*.

Partial least squares-discriminant analyses (PLS-DA) performed for GC/MS, ^1^H NMR, and LTQ Orbitrap MS in ESI^+^ and ESI^−^ data sets (Supplementary Figure 4) revealed in each case two distinct tight clusters, one representing the metabolomes of leaves of inoculated willows and the other that of non-inoculated ones.

Based on the identified biomarkers and applying metabolic networking, the global overview of the willow's leaf metabolic network in response to AMF was obtained (Figure 3). Such network represents an excellent tool for studying metabolism regulation based on large-scale data thanks to the wide range of applications that exist for Cytoscape. Following a reverse genetics approach, the network can also provide useful information on the links between metabolites and proteins, which can be further exploited in systems biology approaches (Ghosh et al., 2011; Aliferis and Jabaji, 2012). Results revealed the general disturbance of willow's metabolism with an increase in the majority of metabolites in mycorrhized trees compared to non-mycorrhized ones. Also, the up-regulation of *α*-linolenic acid metabolism and porphyrin and chlorophyll metabolism is evident in the network (Figure 3).

### Effect of AMF on the willow leaves' chemical composition

**Figure 5:**
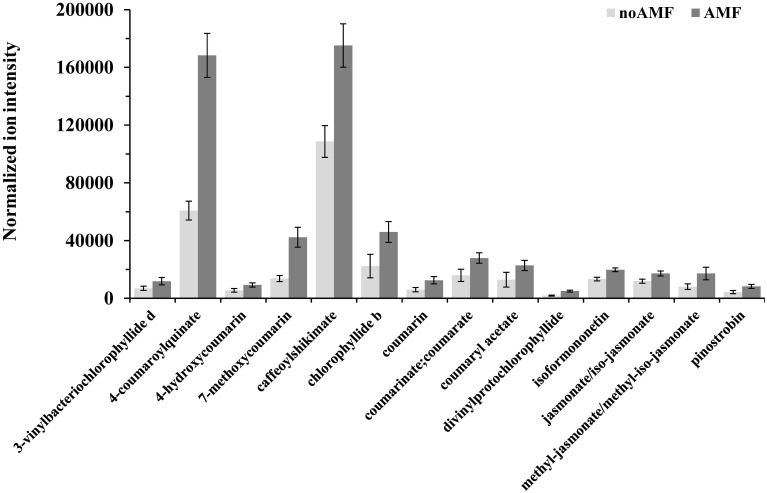
**Normalized ion intensities for representative secondary metabolites that serve as biomarkers of willow leaves in response to *Rhizophagus irregularis* 2 weeks post-inoculation**. Data were recorded performing liquid chromatography-mass spectrometry analysis using an LTQ-Orbitrap acquiring in the positive (ESI^+^) or negative (ESI^−^) electrospray modes. Bars represent standard errors performing the Student's *t*-test (*P* < 0.05).

The high-throughput biological interpretation of large-scale metabolomics data from global profiling experiments is challenging. Nonetheless, the summary of the effect of a treatment on the chemical composition of the biological system being studied could provide a first qualitative overview of the underlying biochemical changes and insights on metabolism regulation in a timely fashion (Aliferis et al., 2014). Here, metabolites belonging to various chemical groups involved in the primary and secondary plant metabolism and symbiosis such as, amino acids, carbohydrates, flavonoids, macrocyclic compounds, phenylpropanoids, and terpenoids, showed a substantial fluctuation 2 weeks following willows' inoculation with AMF (Table 1, Figures 3, 4, 5, and Supplementary Data set 1). Such observation is facilitated by the standardized chemical classification of metabolites in the Willow MetaboLib v.1.0. Metabolites belonging to these groups have important and well-established roles in plant physiology, and exhibit bioactivity. Their potential role during AMF-willow interaction is discussed in more detail in the following sections.

The observed general disturbance of the host's metabolism based on the chemical composition of the leaves is not surprising since symbiosis is a complex and dynamic interaction, whose outcome has to maintain the mutualistic relationship of both partners (García-Garrido and Ocampo, 2002; Cavagnaro, 2008). It also confirms that willow's response or the effect of the AMF on the plant is not localized in the roots, but systemic, which is in accordance with observations in various AMF-plant symbiotic systems (Erb et al., 2009; Schweiger et al., 2014).

### Effect of AMF on the willow's leaf primary metabolism

The direct effect of AMF on the primary metabolism of willow leaves is evident mainly by the fluctuation of their content in carbohydrates and amino acids (Table 1, Figure 3, and Supplementary Data set 1).

The mobilization of willow leaf carbohydrates in response to AMF is in agreement with previous observations (Doidy et al., 2012) and indicative of an underlying operating sugar exchange mechanism between willow and AMF. AMF inoculation resulted in higher levels in the vast majority of monosaccharides and lower levels of sugar alcohols in willow leaves compared to those of non-inoculated ones.

Carbohydrates are products of plants' photosynthetic activity, which generally constitute the bulk of organic material to be translocated through the phloem tissue to the different plant parts (Ainsworth and Bush, 2011; Doidy et al., 2012) and play an important role in plant physiology by regulating gene expression (Koch, 1996). Their content could serve as an indicator of the plant's physiological condition, for example, studies have shown that carbohydrates accumulate in plants under drought conditions (Seki et al., 2007). Also, they are the main nutrient source that the AMF depend on during symbiosis. The observed high levels of carbohydrates in AMF mycorrhized willows can be well attributed to the up-regulation of the porphyrin and chlorophyll biosynthetic pathway (Table 1, Figures 3, 4, and Supplementary Data set 1). The produced monosaccharides are the main carbon source for AMF after their transportation to the roots through monosaccharide transporters (Doidy et al., 2012), as revealed in previous reports (Bonfante and Genre, 2008; Leigh et al., 2009). In addition, the disaccharide α−α-trehalose, whose relative concentration in leaves was significantly increased in response to AMF inoculation, has unique physicochemical properties and is implicated in plant responses to various stimuli (Paul et al., 2008). However, since its concentration in the cells is not clear, trehalose may not work as a protective agent in the biological system being studied.

Sugar alcohols play an important role in plant physiology, including protection against osmolytic and oxidative stress, as well as in plant-pathogen interactions (Williamson et al., 2002). Increased content in sugar alcohols has been reported in plants under drought stress (Seki et al., 2007). The pattern by which the content of a mycorrhized plant in sugar alcohols is altered seems to be species-specific (Schweiger et al., 2014). Based on these observations it can be suggested that the observed decrease of sugar alcohols in AMF-mycorrhized willows is an indication that the plants are under a lower stress level compared to the controls.

The amino acid pool of willow leaves decreased in AMF-inoculated willows (Table 1, Figure 3, and Supplementary Data set 1). Similar findings have been reported during the mycorrhizal symbiosis of *G. mosseae* with *Lotus japonicus* (Fester et al., 2011). However, the effect of AMF on the amino acid content of the host do not exhibit a clear pattern (Hodge and Storer, 2014; Souza et al., 2014), which is indicative of the complexity of the undergoing interactions during symbiosis. Additionally, metabolites involved in the amino acid metabolism were detected in lower amounts in mycorrhized compared to non-mycorrhized plants. This could be attributed to the carbon sink effect induced by AMF and the carbon allocation to the roots where the mycorrhizal interaction takes place.

### Effect of AMF on the willow's leaf secondary metabolism

Symbiosis with AMF caused a general disturbance of willow leaves' metabolism (Table 1, Figures 3, 4, 5, and Supplementary Data set 1). Results revealed up-regulation of key biosynthetic pathways involved in willow responses to biotic and abiotic stresses and indirectly to adaptation. Here, the effect of AMF inoculation on major biosynthetic pathways of willow is discussed.

#### Up-regulation of the phenylpropanoid biosynthetic pathway

The up-regulation of the phenylpropanoid pathway and related metabolites of willow as a main response to AMF (Table 1, Figures 3, 4, and Supplementary Data set 1) is in accordance to previous reports (Morandi, 1996; Pozo et al., 2002). This is a key biosynthetic pathway in plants' physiology, involved, among others, in the biosynthesis of secondary metabolites that play crucial role in responses to stresses (Dixon et al., 2002; Petersen et al., 2010).

Among the identified metabolites, coumaryl acetate, 4-coumaroylquinate, and caffeoyl-shikimate have shown an up-regulation of 1.8, 2.3 and 1.6 fold, respectively, in inoculated plants compared to the controls (Figure 5). Caffeoyl-shikimate is also an intermediate in the lignin biosynthetic pathway (Grassmann, 2005). Lignin is a biopolymer that serves as a matrix around the polysaccharide components of plant cell walls, providing to the latter additional rigidity and structural integrity (Bhuiyan et al., 2009).

Similarly, coumarins and their hydroxy forms increased in response to AMF. Coumarins are well-studied plant secondary metabolites with antimicrobial, antioxidant, and hormonal regulatory properties thus, playing multiple roles in plants' physiology (Bourgaud et al., 2006; Stanchev et al., 2010).

#### Up-regulation of the α-linolenate biosynthetic pathway

The α-linolenate pathway was significantly up-regulated in the presence of AMF (Table 1, Figures 3, 4, and Supplementary Data set 1). Increased levels of OPC6-CoA, trans-2-enoyl-OPC6-CoA, and jasmonates (JAs; jasmonate, JA and iso-JA) were observed in inoculated AMF plants, with the highest increase observed for methyl-jasmonates (methyl-jasmonate/methyl iso-jasmonate, Me-JAs) (2.1 fold) compared to controls (Figure 5).

During mycorrhization, the regulation of hormonal pathways is altered with jasmonic acid (JA) playing a central role during symbiosis (Jung et al., 2012; León Morcillo et al., 2012). To date, it is not clear to what extent shoot-derived JAs contribute to the regulation of mycorrhizal symbiosis in the roots, however, in analogy we expect the JA hormonal pathway to be differentially regulated in the leaves. Evidence on the involvement of the α-linolenate biosynthetic pathway in defense priming during mycorrhization has been recently established (Van Wees et al., 2008; Pozo et al., 2009; Jung et al., 2012). This induction is attributed to its intracellular signal transduction role leading to mediation of secondary metabolite biosynthesis (e.g., phenolic compounds, terpenes, alkaloids, and isoflavonoids) (Schliemann et al., 2008). Strong positive correlation was observed between endogenous concentration of JAs and trichome density, phenylalanine ammonia-lyase (PAL) activity, and phenols concentrations in mycorrhized tomato plants (Kapoor, 2008). This is in accordance with the observed correlation between the increased levels of JAs and metabolites of the phenylpropanoid pathway of willow leaves following inoculation with AMF. Furthermore, it has been reported that biotic and/or abiotic stresses remodel the plant's membrane fluidity by releasing α-linolenate, which in turn plays a protective role in the photosynthetic apparatus (Upchurch, 2008).

#### Up-regulation of the flavonoid, isoflavonoid, and flavone biosynthetic pathways

Inoculation of willows with AMF significantly increased the concentration of the vast majority of features that correspond to flavonoids including those involved in the flavonoid and isoflavonoid biosynthetic pathways of willow leaves (Table 1, Figures 3, 4, and Supplementary Data set 1). The up-regulation of these pathways was among the most interesting findings of the present research. Among the identified components of the two pathways, pinostrobin and isoformononetin significantly increased, 1.9-fold and 1.5-fold, respectively, whereas epicatechin was significantly down-regulated (4.9 fold) in mycorrhized plants (Figure 5).

Flavonoids are major plant secondary metabolites that play a key role in their physiology by protecting them against biotic and abiotic stresses (Pourcel et al., 2007; Dixon and Pasinetti, 2010). Many flavonoids exhibit free radical scavenging and antimicrobial activities (Treutter, 2006; Buer et al., 2010; Cesco et al., 2010; Dixon and Pasinetti, 2010) and in conjugated form, they are also released in the soil as eco-sensing signals for the establishment of suitable symbiotic relationship between plants and rhizobia, AMF or ectomycorrhizal fungi (Shaw et al., 2006; Abdel-Lateif et al., 2012). However, once the AMF-plant association is established, the role of flavonoids in the regulation of mycorrhization is still unclear (Steinkellner et al., 2007). In agreement with our results, flavonoid metabolism in roots and leaves of clover was strongly affected by AMF association, suggesting a strong link between AMF and the regulation of flavonoid metabolism of mycorrhized clover (Ponce et al., 2004).

Focusing on the identified biomarkers, pinostrobin is a potent inducer of antioxidant enzymes (Fahey and Stephenson, 2002) and isoformonetin, a naturally occurring methoxydaidzein, is the product of reaction of daidzein with S-adenosyl-L-methionine (SAM), which is catalyzed by isoflavone-7-O-methyltransferase [EC:2.1.1.150], and its role in plant physiology is not known. Interestingly, isoformonetin has been reported to attract fungal zoospores (Dakora and Phillips, 1996). On the other hand, epicatechin, whose level decreased following AMF inoculation, is the main monomeric unit for proanthocyanidins, a group of polyphenolic compounds with diverse biological and biochemical activities, including protection against predation and pathogen invasion, as well as with an allelopathic function (He et al., 2008).

Finally, in response to inoculation with AMF, the flavonoids rutin and luteolin-7-O-glucoside, which are implicated in the flavone and flavonol biosynthetic pathway, showed a substantial increase of 2.1 and 5 fold, respectively. Both metabolites have been reported as potent antioxidants in *in-vitro* experiments (Süzgeç et al., 2005; Iacopini et al., 2008; Yang et al., 2008). Additionally, luteolin-7-O-glucoside exhibits antimicrobial activity (Chiruvella et al., 2007) and is reported as a plant response to several environmental stresses (Oh et al., 2009; Ahuja et al., 2010).

#### Up-regulation of the porphyrin and chlorophyll biosynthetic pathway

The increase in the vast majority of the identified metabolites involved in the porphyrin and chlorophyll biosynthetic pathway of willow leaves following inoculation with AMF, is another major finding of the present research (Table 1, Figures 3, 4, 5, and Supplementary Data set 1). Such up-regulation of the pathway is likely the cause for the observed higher levels of carbohydrates in the leaves of mycorrhized willows as presented above.

Two of the identified metabolites, chlorophyllide α and chlorophyllide β, are the main precursors of chlorophyll α and β biosynthesis, respectively. The up-regulation of the chlorophyll biosynthetic pathway in AMF-inoculated willows could significantly affect their development, directly and indirectly, through its biosynthetic products and role in defense against pests and pathogens via ROS production. AMF inoculation has been reported to alleviate the content of chlorophyll in plants grown under salinity stress (Sheng et al., 2008; Hajiboland et al., 2010; Latef and He, 2011), which was attributed to the increase in mineral uptake that is mediated via the colonization of roots with AMF.

Not only chlorophylls play a key role in plant growth and are reliable indicators of plant nutrient status, but regulation of their product levels is extremely important being strong photosensitizers generating ROS when they are present in excess (Asada, 2006). In the plant cells, ROS can strengthen the cell walls via cross-linking of glycoproteins (Huckelhoven, 2007) or lipid peroxidation (Montillet et al., 2005). However, it is also evident that ROS are important signaling molecules mediating defense gene activation and controlling various processes including pathogen defense, programmed cell death, and stomatal behavior (Apel and Hirt, 2004; Sharma et al., 2012).

#### Effect of AMF on secondary metabolites with various roles in plant physiology

In addition to metabolites involved in the abovementioned pathways of willow leaves, metabolites with important roles in plant physiology that belong to various chemical groups and are implicated in several biosynthetic pathways, such as, phenolic glucosides and terpenoids were significantly affected by the AMF inoculation (Table 1, and Supplementary Data set 1).

Phenolic glycosides of the *Salix* spp. is their most studied group of metabolites due to their bioactivity and role in plant physiology (Förster et al., 2010; Boeckler et al., 2011), with their levels exhibiting a high seasonal variability (Förster et al., 2010). Here, with the exception of salicortin, all identified phenolic glycosides increased in response to AMF inoculation (Figure 3 and Supplementary Data set 1). These metabolites play an important role in plants' defense against pests and also act synergistically with pathogens on herbivores (Boeckler et al., 2011). This is an indication that mycorrhization of willow could improve its resistance against pests. In addition, willows' phenolic glucosides serve as chemoattractants for herbivorous insects (Kolehmainen et al., 1995).

Additionally, increased levels of terpenoids were recorded in leaves of AMF-inoculated willows compared to those of non-inoculated ones. Terpenoids include metabolites that play a key role in the metabolism of arbuscular mycorrhized roots by regulating major biosynthetic pathways such as, the methylerythritol phosphate pathway and carotenoid biosynthesis (Strack and Fester, 2006). Increased levels of terpenoids are correlated with the production of signaling molecules or protection of root cells against oxidative damage. Several terpenoids function as antioxidants, phytoalexins, and play role in plant defense against pathogens (Grassmann, 2005; Cheng et al., 2007). The induction of these metabolites and their increase following AMF inoculation is expected to have a positive effect on willow's adaptation to stresses.

## Concluding remarks

Here, by the development of a cutting-edge metabolomics/bioinformatics protocol, we investigated in-depth the effects of AMF on willow's leaf metabolism, which could possibly be beneficial for willow when used as a component of integrated phytoremediation strategies. Results unraveled the complexity of AMF-willow interaction, clearly demonstrating beneficial direct and indirect effects on host priming against external stresses as well as by enhancing its growth and productivity. Such interaction is anticipated to provide inoculated plants with a significant advantage over non-inoculated ones by the time that they will be exposed to unfavorable conditions such as, contaminated soil, pests, and pathogens when used as components of a phytoremediation strategy.

## Author contributions

KA and SJ conceived, designed, and executed the experiments. KA, RC, and SJ analyzed the data. KA, RC, and SJ contributed to the writing of the manuscript.

### Conflict of interest statement

The authors declare that the research was conducted in the absence of any commercial or financial relationships that could be construed as a potential conflict of interest.

## Supplementary material

Supplementary Data set 1 **Identified biomarkers of leaves of willow plants 2 weeks following inoculation with *Rhizophagus irregularis* based on GC/MS and LTQ Orbitrap MS analyses**.

Supplementary Figure 1 **Total ion chromatograms (TICs) of willow leaves performing GC/MS analysis**. Representative metabolites are annotated.

Supplementary Figure 2 **Total ion chromatograms (TICs) of willow leaves performing LC/MS analysis using an LTQ Orbitrap Classic analyzer in the positive (ESI^+^) and negative (ESI^−^) electrospray modes**.

Supplementary Figure 3 **^1^H NMR spectra of willow control (A) and mycorrhized (C) willow leaves**. Corresponding partial least squares (PLS) coefficient diagram with values of scaled and centered PLS regression coefficients (CoeffCS) is displayed **(B)**. Representative metabolites are annotated.

Supplementary Figure 4 **Partial least squares-discriminant analyses (PLS-DA) PC1/PC2 score plots of GC/MS, ^1^H NMR and LTQ Orbitrap MS (ESI^+^ and ESI^−^) metabolite profiles of control (■) and willow leaves 2 weeks following inoculation with the AMF *Rhizophagus irregularis* ()**. The ellipse represents the Hotelling T^2^ with 95% confidence interval. Five (5) biological replications were used per treatment and one quality control sample (QC) [Q^2^_(*cum*)_; cumulative fraction of the total variation of the X's that can be predicted by the extracted components, *R*^2^*X* and *R*^2^*Y*; the fraction of the sum of squares of all *X*'s and *Y*'s explained by the current component, respectively. *PCs*, principal components].
