# Supplementary figures and images for: Metabolic responses of willow (Salix purpurea L.) leaves to mycorrhization as revealed by mass spectrometry and 1H NMR spectroscopy metabolite profiling
Source: Front Plant Sci. 2015 May 18;6:344. doi: 10.3389/fpls.2015.00344 (PMC4434919; doi:10.3389/fpls.2015.00344)

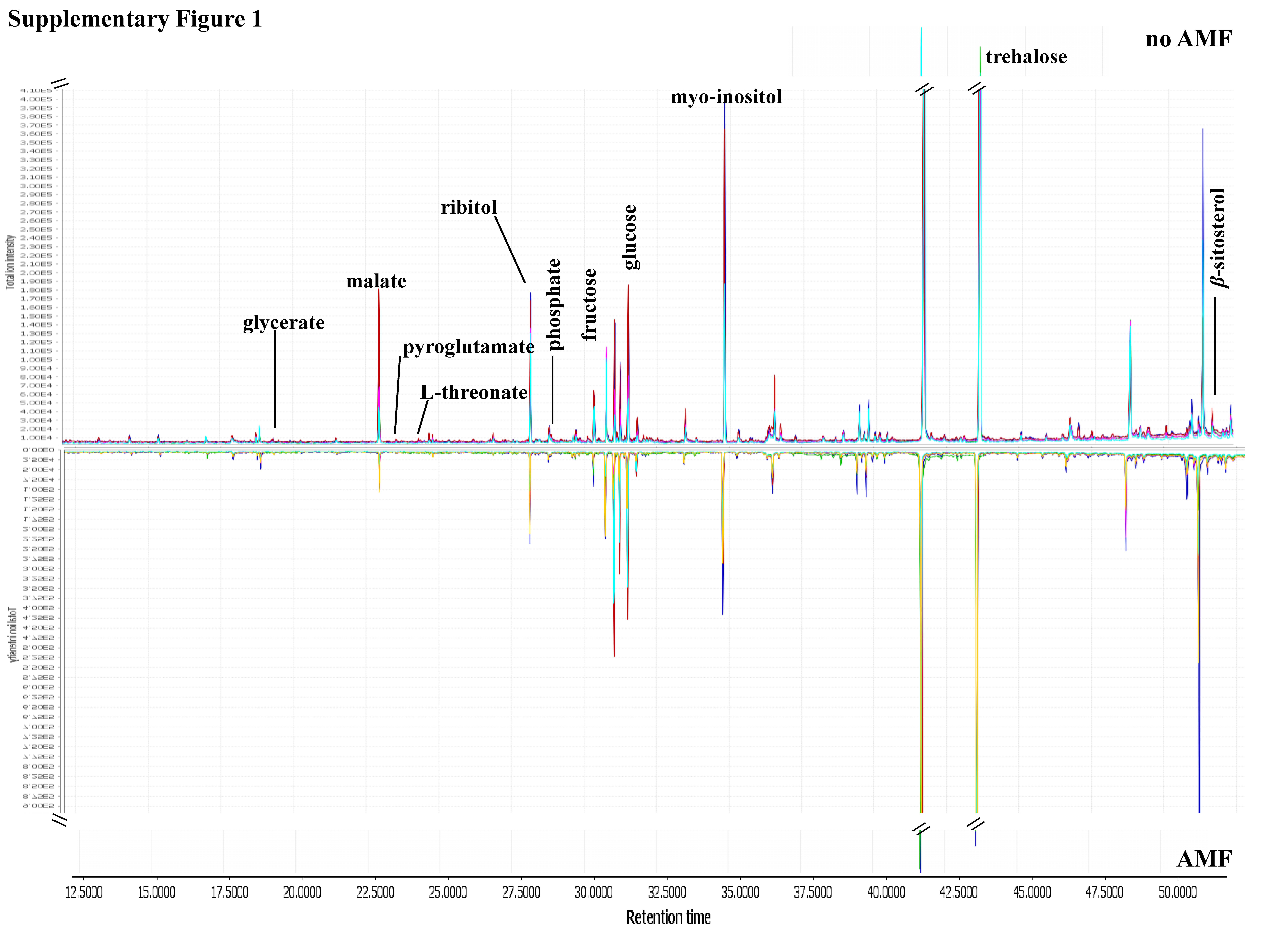

Supplement: Supplementary Figure 1 — Total ion chromatograms (TICs) of willow leaves performing GC/MS analysis. Representative metabolites are annotated. [file Image1.JPEG]

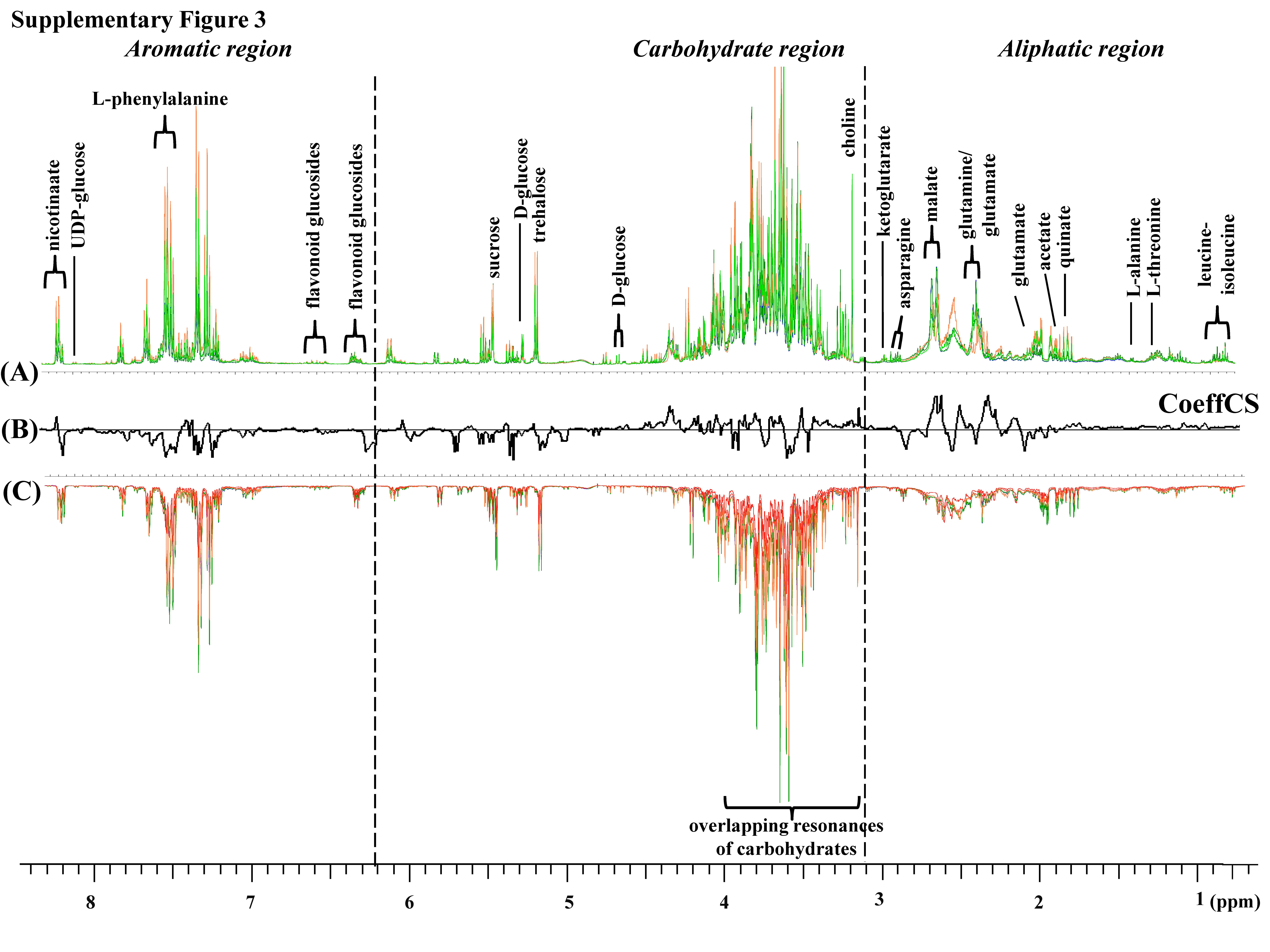

Supplement: Supplementary Figure 3 — 1H NMR spectra of willow control (A) and mycorrhized (C) willow leaves. Corresponding partial least squares (PLS) coefficient diagram with values of scaled and centered PLS regression coefficients (CoeffCS) is displayed (B). Representative metabolites are annotated. [file Image3.JPEG]

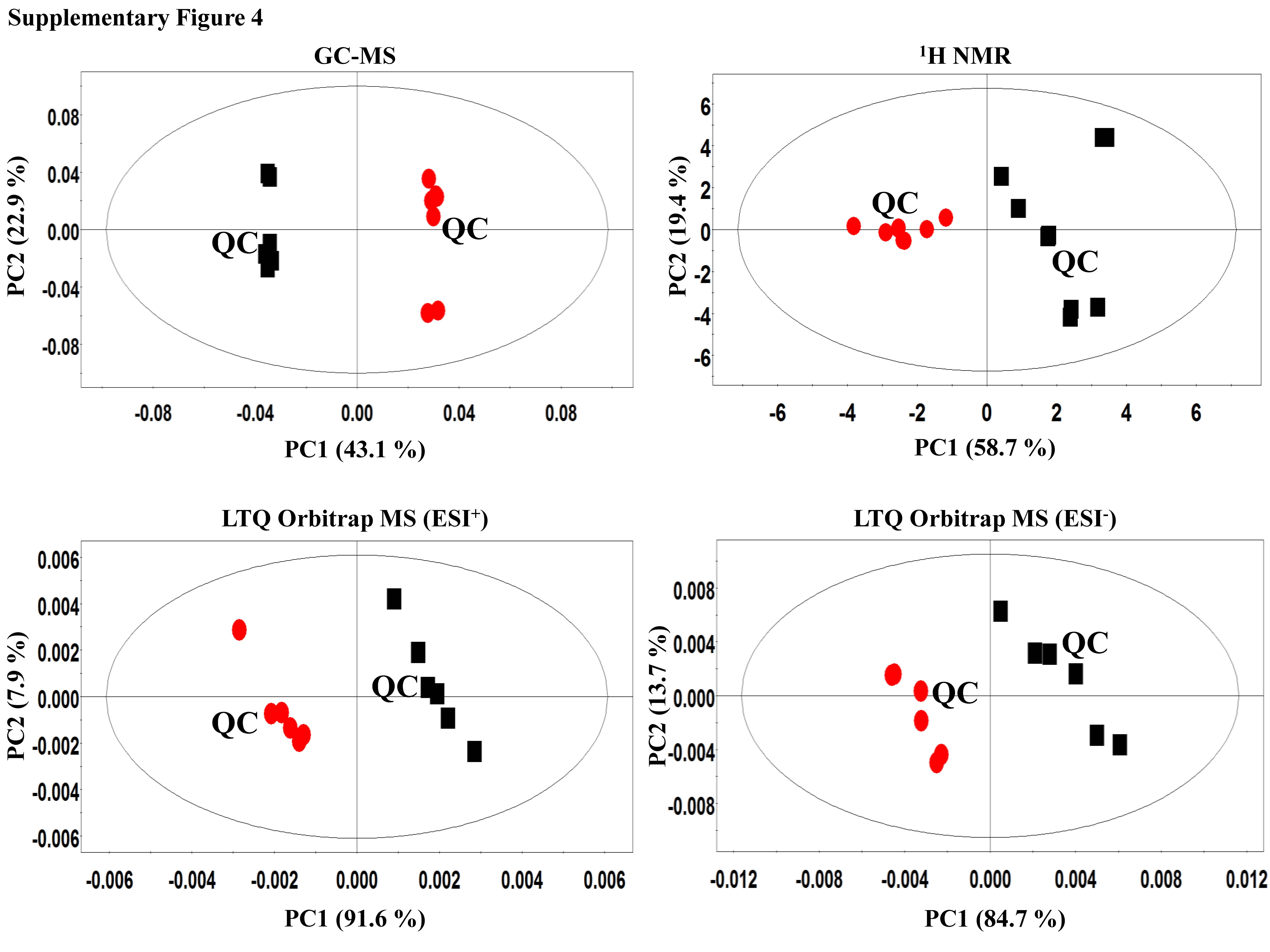

Supplement: Supplementary Figure 4 — Partial least squares-discriminant analyses (PLS-DA) PC1/PC2 score plots of GC/MS, 1H NMR and LTQ Orbitrap MS (ESI+ and ESI−) metabolite profiles of control (■) and willow leaves 2 weeks following inoculation with the AMF Rhizophagus irregularis (). The ellipse represents the Hotelling T2 with 95% confidence interval. Five (5) biological replications were used per treatment and one quality control sample (QC) [Q2(cum); cumulative fraction of the total variation of the X's that can be predicted by the extracted components, R2X and R2Y; the fraction of the sum of squares of all X's and Y's explained by the current component, respectively. PCs, principal components]. [file Image4.JPEG]
