# Supplementary material for: Metabolic responses of willow (Salix purpurea L.) leaves to mycorrhization as revealed by mass spectrometry and 1H NMR spectroscopy metabolite profiling
Source: Front Plant Sci. 2015 May 18;6:344. doi: 10.3389/fpls.2015.00344 (PMC4434919; doi:10.3389/fpls.2015.00344)
Supplement: Supplementary file 6 [file Table1.DOCX]

**Supplementary Table 1.** Gradient program for LC/MS analysis using an LTQ Orbitrap Classic analyzer operating in positive electrospray mode (ESI^+^).

| Time (min) | H_2_O:formate 0.1% (v/v) (%) | Acetonitrile (ACN):formate 0.1% (v/v) (%) | μL min^-1^ |
| --- | --- | --- | --- |
| 0.0 | 95 | 5 | 190 |
| 0.1 | 95 | 5 | 450 |
| 3.9 | 95 | 5 | 450 |
| 4.0 | 95 | 5 | 190 |
| 49.0 | 65 | 35 | 190 |
| 51.0 | 25 | 75 | 190 |
| 56.0 | 25 | 75 | 190 |
| 58.0 | 95 | 5 | 190 |
| 64.0 | 95 | 5 | 190 |
