# Supplementary material for: Metabolic responses of willow (Salix purpurea L.) leaves to mycorrhization as revealed by mass spectrometry and 1H NMR spectroscopy metabolite profiling
Source: Front Plant Sci. 2015 May 18;6:344. doi: 10.3389/fpls.2015.00344 (PMC4434919; doi:10.3389/fpls.2015.00344)
Supplement: Supplementary file 8 [file Table3.DOCX]

| Parameter | Value |
| --- | --- |
| Capillary Temp (C): | 300 |
| APCI Vaporizer Temp (C): | 150 |
| Sheath Gas Flow (): | 25 |
| Aux Gas Flow (): | 10 |
| Sweep Gas Flow (): | 0 |
| Source Type: | HESI |
| Injection Waveforms: | Off |
| Ion Trap Zoom AGC Target: | 4000 |
| Ion Trap Full AGC Target: | 30000 |
| Ion Trap SIM AGC Target: | 10000 |
| Ion Trap MSn AGC Target: | 10000 |
| FTMS Injection Waveforms: | Off |
| FTMS Full AGC Target: | 500000 |
| FTMS SIM AGC Target: | 50000 |
| FTMS MSn AGC Target: | 400000 |
| Source Voltage (kV): | 4 |
| Source Current (uA): | 100 |
| Capillary Voltage (V): | 5 |
| Tube Lens (V): | 60 |

**Supplementary Table 3.** Settings for LC/MS analysis using an LTQ Orbitrap Classic analyzer operating in positive electrospray mode (ESI^+^).
