# Supplementary material for: Metabolic responses of willow (Salix purpurea L.) leaves to mycorrhization as revealed by mass spectrometry and 1H NMR spectroscopy metabolite profiling
Source: Front Plant Sci. 2015 May 18;6:344. doi: 10.3389/fpls.2015.00344 (PMC4434919; doi:10.3389/fpls.2015.00344)
Supplement: Supplementary file 11 [file Table6.DOCX]

**Supplementary Table 6.** Settings for the software Sieve v.2.0 for chromatogram analysis in negative electrospray mode (ESI^-^)

| Parameter | Value |
| --- | --- |
| RT Width (min) | 1 |
| RT Stop (min) | 59.5 |
| RT Start (min) | 2.53 |
| MZ Width PPM (ppm) | 5 |
| MZ Stop (Da) | 1200 |
| MZ Start (Da) | 90 |
| PR Max Charge () | 3 |
| Background SN | 3 |
| Maximum Frames | 120000 |
| Threshold | 100000 |
